# *In Vitro* and *Ex Vivo* Selection Procedures for Identifying Potentially Therapeutic DNA and RNA Molecules 

**DOI:** 10.3390/molecules15074610

**Published:** 2010-06-28

**Authors:** Soledad Marton, José A. Reyes-Darias, Francisco J. Sánchez-Luque, Cristina Romero-López, Alfredo Berzal-Herranz

**Affiliations:** Instituto de Parasitología y Biomedicina “López-Neyra”, CSIC, P.T. Ciencias de la Salud, Av. del Conocimiento s/n, Armilla, 18100 Granada, Spain; E-Mails: smarton@ipb.csic.es (S.M.); joanreda@hotmail.com (J.A.R.D.); kiko@ipb.csic.es (F.J.S.L.); cristina_romero@ipb.csic.es (C.R.L.)

**Keywords:** *in vitro* selection, SELEX, aptamer, ribozyme, therapeutic nucleic acids

## Abstract

It was only relatively recently discovered that nucleic acids participate in a variety of biological functions, besides the storage and transmission of genetic information. Quite apart from the nucleotide sequence, it is now clear that the structure of a nucleic acid plays an essential role in its functionality, enabling catalysis and specific binding reactions. *In vitro* selection and evolution strategies have been extremely useful in the analysis of functional RNA and DNA molecules, helping to expand our knowledge of their functional repertoire and to identify and optimize DNA and RNA molecules with potential therapeutic and diagnostic applications. The great progress made in this field has prompted the development of *ex vivo* methods for selecting functional nucleic acids in the cellular environment. This review summarizes the most important and most recent applications of *in vitro* and *ex vivo* selection strategies aimed at exploring the therapeutic potential of nucleic acids.

## 1. Introduction

Nucleic acids, particularly RNA, are extremely versatile molecules. Apart from their role as carriers of genetic information they can also express a phenotype, e.g., they may show a catalytic activity, have a specific binding function, or have the capacity to recruit specific molecules. 

The appearance of sequence variation in a nucleic acid can, in some cases, provide an advantage to an organism: a key feature in natural evolution. Over the last 20 years, advances in molecular biology and biotechnology have seen the development of methods that allow the effect of such naturally arising variation to be mimicked in the laboratory. It was in the 1960s when Spiegelman’s group first observed evolution *in vitro* [1]. These authors reported that changes in the RNA genome of the Qβ bacteriophage during replication led to the formation of RNA molecules that were more efficiently copied by the viral replicase. These genomes lacked unnecessary sequences and were synthesized at a greater rate. However, these finding fell into oblivion until 1990, when the great potential of *in vitro* selection and evolution techniques was reported by three independent groups [2,3,4,5]. Since then, numerous authors have used these technologies to study the chemical and catalytic properties of nucleic acids (for further information see [6,7,8,9,10,11,12,13]. They have also helped uphold the RNA world hypothesis, and it seems likely that they may soon have applications in biomedicine. Indeed, new selection methods – known as *ex vivo* selection procedures – are now being used to identify molecules that target viruses, subcellular fractions and even whole cells. These techniques overcome some of the limits imposed by *in vitro* technology and provide new environments and conditions in which to explore the properties and functions of nucleic acids. This review highlights the most recent advances in *in vitro* and *ex vivo* selection procedures for nucleic acids, and discusses their potential application in biomedicine.

## 2. General Principles of *in Vitro* Selection Methods

**Figure 1:**
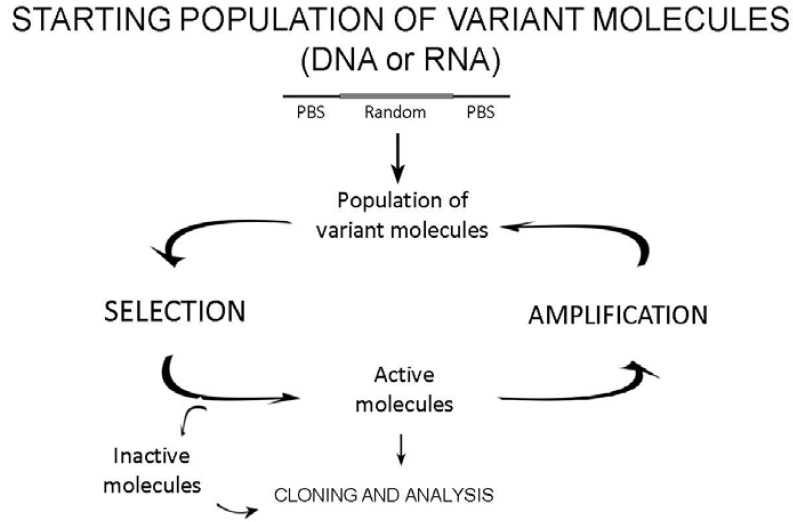
General scheme of *in vitro* selection procedures. The starting population of variant molecules enters in the selection cycle. Sequence variants are separated into active, those that satisfy selection criteria, and inactive molecules. Active molecules are selectively amplified by PCR resulting in the production of a new pool of molecules that can be the input of a new selection cycle. An additional reverse transcription and *in vitro* transcription steps are required, before and after PCR, respectively, when the selection is performed on RNA molecules. Alternatively, active and inactive resulting pools can be cloned and analyzed.

*In vitro* selection strategies have been used to select nucleic acids with a large variety of properties. Although each strategy differs according to the feature or phenotype sought, all *in vitro* selection methods follow the same three-step pattern (Figure 1).

### 2.1. The design of starting variant populations

Genetic variability is introduced into the system (generally by chemical synthesis) to yield nucleic acid populations, the heterogeneity of which is determined by fixing the number of nucleotides to be mutagenized and the mutation rate per nucleotide. In most cases, completely random synthesis involving a fixed number of nucleotides yields a starting population of variant molecules that, *a priori*, differ only in the sequence and the structure of the variable region. Constant sequences, or primer binding sites (PBS), flanking the variable region are incorporated during the design of the starting population to facilitate the amplification of desired molecules, although this limits the structural diversity of the RNA populations to specific conformations [14]. Different approaches have been tried to minimize the effect of PBSs, e.g., the addition of customized primers or adapters by ligation before amplification, and their removal prior to the selection step [15,16,17]. 

### 2.2. Selection

The selection strategy needs to be specifically designed according to the phenotype sought. The initial pool of variants usually contains very few active molecules corresponding to the desired phenotype, and their enrichment can only be made possible by properly designing the selection step. The selection of inactive molecules may also be important since the analysis of these molecules can provide very valuable information on the sequence and structural requirements of active molecules [18]. *In vitro* selection strategies have been widely used for the selection of nucleic acids capable of catalyzing specific chemical reactions, *i.e.*, DNAzymes and ribozymes [19]. These strategies have also been successfully used to identify DNA and RNA molecules with affinity for a specific ligand (*i.e.*, aptamers) [3], being known as SELEX, which stands for systematic evolution of ligands by exponential enrichment [5].

### 2.3. Amplification of active molecules

Variants that show the required phenotype need to be replicated to ensure their passage into the next generation and therefore their persistence in the population. Specific primer binding sites are used to amplify the selected molecules. When necessary a RNA polymerase promoter is incorporated at the 5’ end of the PBS during the amplification step.

In addition to these general steps, the amplified molecules may require additional manipulations prior to their introduction into the next round of selection When the selected molecule is ssDNA, both DNA strands must be separated and the positive one isolated, e.g., by incorporating a biotinylated residue into the unwanted strand [20,21] via asymmetric PCR [22].

As a result of the described selection cycle, a population enriched in the sought-after molecules (but not composed of them entirely) is produced; a new selection cycle can then be undertaken. By iteratively executing the process of selection and amplification the complexity of the original population is reduced and enriched with candidates of interest. During this process, the stringency of selection can be increased to achieve the isolation of the molecules with the desired phenotype. 

## 3. *Ex Vivo* Selection

The great advances made in *in vitro* selection procedures over recent decades have helped us improve our knowledge of the plasticity of nucleic acids and their potential applications as therapeutic tools. Indeed, such have been the advances that selection strategies within living cells are now contemplated. These new methodologies are named according to the specific procedure employed in each method, e.g., *ex vivo*, *in vivo*, *in cell* selection, or Cell-SELEX *etc*. These are here all referred to as *ex vivo* selection strategies.

*Ex vivo* selection methodologies follow the general pattern described above for *in vitro* techniques. These systems have mainly been used in the isolation of DNA and RNA molecules that interfere with the activity of a target molecule. When the expression of an oligonucleotide leads to a desired cell phenotype, it allows for the selection of these cells and the subsequent isolation of the oligonucleotide itself. Following these principles a strategy was used to identify a transcriptional activator regulated by TMR (tetramethylrosamine) [23]. For this purpose, a chimera was constructed by tethering a TMR aptamer to the transcriptional activator for MS2 protein (also known as N40-26) [24]. Variability was introduced by randomizing seven nucleotides within the linker region of the two RNA domains. Selection was performed in yeast cells containing a construct coding for the HIS3 and LacZ genes under the control of the LexA operator, and expressing a LexA-MS2 coat protein fusion. This is a hybrid protein that binds to the operator and to the N40-26 MS2 RNA hairpin present in the RNA construct. Only cells expressing an active transcriptional activator were capable of growth in the absence of histidine, and were the only ones capable of expressing β-galactosidase. The stringency of the selection process was increased by the presence of varying amounts of a competitive inhibitor of the His3p activity. Analysis of the selected yeast clones revealed specific RNA sequences that responded to TMR enhancing the transcription activator effect [23]. 

The selection of an artificial ribosome switched on or off via the external addition of a small molecule to the growth medium deserves special mention. The *Escherichia coli* 16S ribosomal RNA was fused to an aptazyme, a ribozyme capable of binding thiamine pyrophosphate (TTP) through an aptamer domain. The binding of TTP activates the ribozyme, reducing gene expression by cleaving the 16S ribosomal RNA and thus working as a riboswitch. This allows for the identification and selection of specific *E. coli* colonies according to the phenotype expressed [25]. The TTP riboswitch was also included in the 5`UTR region of a reporter gene, 30 nt upstream of the Shine-Dalgarno sequence. This linker between these elements was randomized and *E. coli* colonies selected depending on the expression of the reporter gene in response to TTP [26].

*Ex vivo* selection methods in cells are limited by the number of sequences that can be studied, this number being determined by the number of available cells. Another problem is the possibility of there being more than one sequence variant per cell, which could lead to many false positive. Ellington’s group developed a very interesting strategy that allowed the direct selection of active molecules instead of selecting cell clones [27]. The cleavage products of the autocatalytic ribozymes synthesized in the cell nucleus were extracted via hybridization to a biotinylated oligonucleotide, allowing the direct identification of active molecules.

The selection of nucleic acids against whole cell targets has been successfully used to select nucleic acids that target cell surface receptors. This technology also known as cell-SELEX could have a role to play in the treatment of cancer. For most types of cancer cell there is a shortage of highly specific surface markers that can be used with diagnostic and therapeutic intent. Aptamers generated from whole living cells are the optimal molecular probe for characterizing target cells at the molecular level. When bound to the membrane receptors of cell lines they provide an effective means of identifying disease markers.

## 4. Post-selection Modifications

The pharmacokinetic and pharmacodynamic properties of a nucleic acid, and its resistance to nucleases, all condition its effectiveness as a therapeutic molecule. After selection, the most effective molecules can be modified to improve their nuclease resistance as well as their affinity for their targets, their cellular uptake and selectivity. A great diversity of post-selection modifications has been described. Only those of interest for therapeutic purpose are discussed here. These include modifications of oligonucleotide size and sequence, and mainly certain chemical modifications (for a review see [28,29]. 

**Figure 2:**
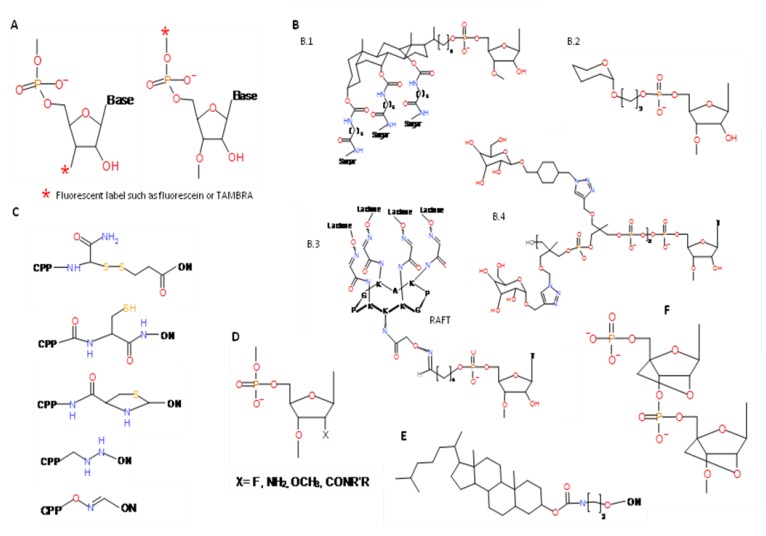
Most relevant chemical modifications which significantly contribute to improve pharmacokinetic and pharmacodynamic properties of aptamers. A) Flourescent label. B) Most common glycoside modifications: B.1, Trigalactosylated tetrahydroxycholate conjugate, B.2, Galactose conjugate, B.3, Multiple carbohydrates can be attached through a cyclopeptide scaffold like RAFT: radioselectively addressable functionalized template. B.4, Polyamine conjugate with two manosyl and two galactosyl residues. C) Most commonly used covalent linkers to conjugate cell penetrating peptides (CPP) and an oligonucleotide (ON) chain. D) 2’-modified ribonucleosides. E) Cholesterol conjugate. F) Locked nucleotides (LNA).

Advances in chemical synthesis have allowed the production of conjugates that combine an oligonucleotide sequence with compounds such as fluorophores, peptides, carbohydrates and lipids (Figure 2). These modified oligonucleotides show advantageous properties with respect to the native form. The ligands are usually linked to the 5’ or 3’ termini, which are the most accessible regions for chemical conjugation reactions; in addition, any disruption of the nucleic acid’s folding and functional properties are minimized [30]. Conjugations at the 2’ position of ribose or involving the internucleotidic phosphodiester bonds are also possible. Fluorophore conjugation is already used in clinical diagnosis, e.g., in fluorescence *in situ* hybridization (FISH) and molecular beacons. FISH can detect specific genes in cells, while molecular beacons act like switches, emitting fluorescent light when bound to their target sequence. Cell uptake has been improved by the conjugation of oligonucleotides to peptides capable of translocating them across the cell membrane by a non-receptor-mediated endocytotic mechanism. Some of the most frequently used peptides are residues 43-58 of the third helix of the antennapedia homeodomain (penetratin), the highly arginine/lysine rich region of the HIV-1 Tat protein, the hydrophobic signal peptide, the nuclear localization sequence (NLS), and transportan [31]. Besides improving cellular uptake, peptide-oligonucleotide conjugates show increased stability to nucleases degradation and enhanced binding [32]. Carbohydrate-oligonucleotide conjugates (COCs) have similar applications. In addition they confer cell or tissue specificity by their binding to sugar receptors (*i.e.*, lectins) present at the cell surface capable of recognizing and internalizing (by endocytosis) glycoproteins bearing specific carbohydrates moieties [33]. Lipophilic oligonucleotide conjugates (LOCs), such as cholesterol, reduce the hydrophobic character of the oligonucleotide, and some bind to blood lipoprotein carriers. Lipophilic oligonucleotides are also used for designing supramolecular assemblages such as micelles, vesicles and liposome networks [34].

The ability of certain RNA polymerases to incorporate modified nucleotides to the growing chain, such as the 2’-modified ribonucleosides, makes it possible to use the selection procedure with populations of chemically modified oligonucleotides [35,36]. Chemical modifications of the 2’ hydroxyl group of RNA, such as 2’ fluoro, amino, methoxy and amido modifications, are noteworthy for their potential therapeutic applications since they increase RNA stability, conferring greater resistance to nucleases. Another important modification for therapeutic purposes is the use of locked nucleotides (LNAs) in the nucleotidic chain. LNAs contain a methylene link between the 2’-O and 4’-C of the ribose ring which locks the sugar moiety in the 3’ endo conformation [37]. This generates the most stable hybrids ever characterized, with a ΔT_m_ of +3 to +10 per LNA residue upon binding to DNA and RNA respectively, thus conferring nuclease resistance [38,39]. The introduction of LNA modifications into *in vitro* selection techniques has so far been restricted to post selection incorporation (for a review see [40]). Nevertheless, it has recently been shown that locked nucleotides can be incorporated enzymatically into both DNA and RNA [41,42,43]. 

Spiegelmers (Spiegel = mirror in German), unnatural but biostable L-forms of D-aptamers, were developed to allow oligonucleotides to escape nuclease attack. Naturally occurring proteins are L-chiral, therefore natural nucleases digest D-oligonucleotides while L-nucleosides escape this fate. To obtain spiegelmers, natural oligonucleotides are used during the selection cycle against unnatural D-proteins that mirror the natural structure of the L-target [44,45]. The selection process yields L-form aptamer sequences that by virtue of the law of symmetry recognize their natural target. Spiegelmers have been selected against peptide hormones such as gonadotropin-releasing hormone (GnRH) [46], and ghrelin, an endogenous ligand for growth hormone secretagogue receptor 1a [47]. Both have a neutralizing effect *in vivo* against these hormones after systemic administration. 

## 5. Therapeutic Applications of Nucleic Acid Selection Procedures

*In vitro* selection strategies have been extensively and successfully used to characterize known ribozymes and DNAzymes, and to isolate new catalytic nucleic acids with unsuspected activities. In fact, the first observation of a DNA molecule catalyzing a chemical reaction (DNAzyme) was made when using an *in vitro* selection strategy [48]. Although ribozymes and DNAzymes have been extensively assayed as potential therapeutic agents, and different clinical trials have already tested their efficiency against various diseases [49,50,51,52], very few reports have described the direct application of *in vitro* selection strategies in the development of potentially therapeutic catalytic nucleic acids. Ellington’s group recently described a procedure aimed at identifying cleaving ribozymes active within the cell milieu, but this has not yet been used with therapeutic intent [27]. Most of the work referred to herein describes the use of *in vitro* and *ex vivo* selection strategies for the identification of aptamers of therapeutic potential. Assays with catalytic nucleic acids engineered by so-called ‘rational design’ are beyond the scope of this review. 

## 6. Aptamers as Therapeutic Agents

The idea that aptamers can modulate the activity of target proteins emerged from basic studies of viruses. In the 1980s, research on HIV and adenovirus led to the discovery that these viruses contain several structural RNA domains that bind to viral or cellular proteins with high affinity and specificity. Not surprisingly, functional analyses of these viral RNA ligands demonstrated that the viruses had evolved these aptamers either to modulate the activity of proteins essential for their replication [53] or to inhibit the activity of proteins involved in cellular antiviral responses [54]. The first study performed to determine whether an RNA aptamer could be used to inhibit the activity of a pathogenic protein was published in 1990. This work reported that the TAR aptamer, evolved by HIV to recruit viral and cellular proteins to viral transcripts, could be turned against the virus to inhibit its replication [55]. The *in vitro* aptamer selection strategies developed during the 1990s prompted the idea of Sullenger’s group that therapeutic aptamers might be possible. Several such aptamers have now completed various stages of preclinical development, and a number of others are currently being tested clinically (Table 1). Indeed, one aptamer is already on the market as a therapeutic drug

**Table 1:** Aptamers obtained by *in vitro* selection currently in clinical trials or approved for their use as therapeutic drugs. Updated June 2009.

Therapeutic target	Aptamers	Type	Disease indication	Clinical status	Reference
**VEGF**	Macugen^TM^ (Pegaptanib Sodium)	2’-Fluoro- 2’-O-methyl RNA+PEG	Macular degeneration	Market	[57,58]
**Von Willebrand factor**	ARC1779	DNA/RNA+PEG	Thrombotic microangiopathy Adjunct to carotid endarterectomy	Phase 2 Phase 2	[59,60]
**Factor IXa**	REG-1 (RB006 aptamer + RB007 antidote)	RB006 2’-Fluoro RNA+PEG and RB007 2’-O-methyl RNA	Coronary artery bypass Percutaneous coronary intervention	Phase 2 Phase 2	[61,62,63]
**Nucleolin**	AS1411	DNA	Acute myeologenous leukemiaRenal cell carcinoma	Phase 2 Phase 2	[64,65,66]
**PDGF-b**	E10030	DNA	Macular degeneration	Phase 1	[67,68]
**Complement factor 5**	ARC1905	2’-Fluoro RNA	Macular degeneration	Phase 1	[69]
**Thrombin**	NU172	DNA	Coronary artery bypass	Phase 1	[70,71,72]

VEGF = vascular endothelial grow factor; PDGF-b = Platelet-derived growth factor B-chain; PEG = polyethylene glycol.

Several modifications of the general selection process scheme have been described in order to achieve different goals. For example, the toggle-SELEX strategy is used for the selection of potentially therapeutic nucleic acids [56]. Toggle-SELEX was designed for the isolation of aptamers with a broad range of specificities for closely related targets, such as the homologous proteins of different species. These aptamers were obtained by performing the selection procedure for related targets (*i.e.*, homologous proteins) in alternating cycles. Such alternation ensures that the RNA or DNA variants resulting from selection will bind to both proteins, most likely to domains conserved between them. Sullenger’s group described an *in vitro* selection strategy in which RNA aptamers that bind both human and porcine thrombin were selected by "toggling" the protein target between the human and porcine forms in successive rounds of selection [56]. This yielded a family of aptamers, all of which bound both thrombin types with high affinity. Toggle-25, a characteristic member, inhibited two of thrombin's most important functions: plasma clot formation and platelet activation [56]. This strategy could facilitate the isolation of ligands with properties required for gene therapy or other therapeutic or diagnostic applications.

### 6.1. Aptamer-based anti-degenerative disease agents

**Figure 3:**
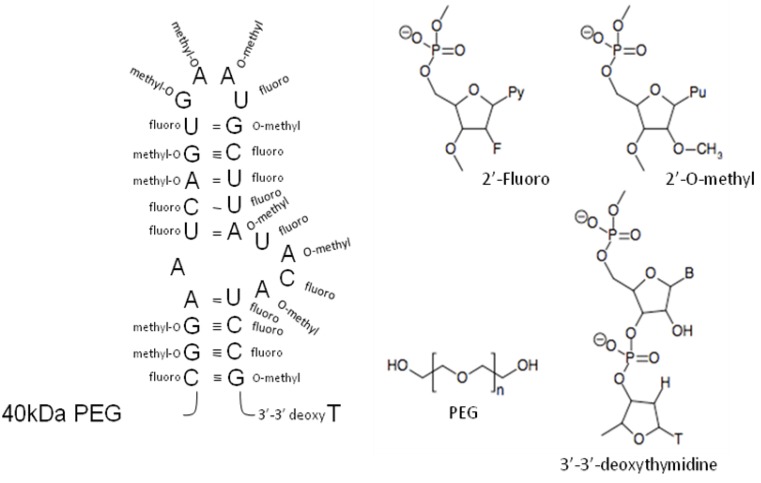
Pegaptanib aptamer. Proposed secondary structure model of the pegaptanib aptamer is indicated on the left. All pyrimidines were 2’-fluoromodified during selection procedure. All purines of the aptamer were 2’-*O-*methyl-modified after the selection. Additionally 40 kDa polyethylene glycol was added in 5’-end and an inverted 3’-3’-deoxythymidine cap in 3’ the end. Chemical structure of indicated modifications is shown on the right [57,74].

To date, the only aptamer approved by the FDA [73], known as Pegaptanib or Macugen, was approved in December 2004 for the treatment of wet type age-related macular degeneration (AMD). This aptamer binds to vascular endothelial growth factor, VEGF_165_ [57,74], the main isoform of a family of growth factors involved in promoting blood vessel development and maintenance via tyrosine kinase receptor signaling. VEGF_165_ is also involved in several pathological processes such as AMD, diabetic retinopathy and cancer [75]. A Phase II clinical trial to evaluate the use of this aptamer in the fight against diabetic retinopathy is currently underway [76]. The selection procedure involved a 2’-fluoro-pyrimidine (2’-FY)-modified RNA pool. Additional modifications were made after selection by adding 2’-O-methyl (2’-MR) to all purine residues of the aptamer except two, by adding a 3’ cap, and by adding polyethylene glycol (PEG; 40 kD) to the 5’ end (Figure 3). The Macugen aptamer binds to the heparin-binding domain of VEGF_165_ [75,77] and efficiently inhibits the growth of blood vessels [57,74]. This agent is of particular interest with respect to preventing tumor angiogenesis. 

Different aptamers are currently being tested for the treatment of other degenerative diseases. Niu’s group attempted to interfere with the function of the GluR2 AMPA receptor associated with cerebral ischemia and amylotrophic lateral sclerosis [78]. A selected aptamer known as AN58 acts as a glutamate antagonist preventing glutamate-induced activation of the cationic channel. Interestingly, the aptamer adopts two mutually exclusive non-interchangeable isoforms that are both necessary for proper inhibition to occur [79]. Recently, new RNA aptamers have been isolated that bind to the Nogo-66 neural receptor as antagonists of myelin-derived ligands. Nogo-66 signaling blocks neurite outgrowth, but the binding of the aptamers allow axon growth in rat ganglion cells *in vitro* [80]. This aptamer is of special interest in the search for agents that aid neural repair, e.g., after spinal cord trauma.

Aptamers can also be targeted against disease-causing proteins such the scrapie isoform of the prion protein (PrP^SC^). A 2’-FY RNA aptamer known as SAF-93 [81] has been shown to prevent its aggregation in cell free systems, while a 2’-amino-2’-deoxypyrimidine-modified aptamer known as DP7 slows down its aggregation in neuroblastoma cells [35]. Both aptamers target the same conserved region involved in prion interactions.

### 6.2. Anti-inflammatory aptamers

Neutrophil elastase (hNE) is involved in the pathogenesis of inflammatory diseases such as acute respiratory distress syndrome (ARDS), septic shock, emphysema and arthritis, as well as ischemia-reperfusion injuries [82]. A covalent inhibitor of hNE, a diphenyl phosphate derivative of valine, has been coupled to an RNA library to enhance its binding to hNE [83]. After *in vitro* selection, an RNA aptamer conjugated with DNA:valP (RNA10.11:DNA:valP) was isolated that binds hNE with high affinity. The bound molecule, unlike the aptamer RNA 10.11 or DNA:valP alone, also inhibits lung inflammation in an *ex vivo* rat model of ARDS [83]. More efficient inhibitors of hNE were obtained from a valyl phosphonate:DNA pool [84]. After selection, aptamer inhibitor ED45 inhibited hNE formation two orders of magnitude greater than RNA.10.11:DNA:valP [83]. A truncated DNA aptamer version named NX21909, composed of two annealed DNA oligonucleotides, was tested in a rat model of lung inflammation and was found to inhibit neutrophil infiltration by 53% at a dose of 40 nmol [85].

### 6.3. Anti-immunoglobulin E aptamers

Immunoglobulin E (IgE) plays an important role in protecting mammals from parasites [86]. However, its overproduction due to exposure to environmental antigens can result in allergies, atopic dermatitis and allergic asthma [87]. DNA selection was performed against human IgE to produce aptamers that bind it with high affinity [88]. These aptamers inhibited the binding of IgE to its receptor Fc*ε*RI, and also prevented IgE-mediated cellular degranulation in the serum of patients with allergy to grass pollen. In patients exposed to grass pollen extract, the IC50s for the DNA aptamers were 2–6 μM, but when triggered by anti-IgE antibodies they reached 200–300 nM. These DNA aptamers represent a novel class of IgE inhibitors that may prove useful in the fight against allergic diseases.

### 6.4. Aptamer-based therapy against cancer

Cancer has been one of the diseases most targeted by aptamers. Alteration of the signaling pathways results in the escape of tumor cells from the control of cell division and apoptosis. The formation of metastases is also promoted. All of these processes have been the target of aptamers. The tyrosine kinase receptors (TKR) has been targeted by two aptamers: RET (rearranged during transfection) and HER 3 (human epidermal growth factor receptor). The RET aptamer, D4, a 2’-FY aptamer, was isolated by *ex vivo* selection against the extracellular mutant RET^C634y^ receptor [89]. Briefly, an RNA population was incubated against a parental PC12 cell line and variants expressing different RET mutants as a negative selection step. The unbound fraction of RNA molecules was recovered and incubated against PC12 cells expressing the recombinant RET^C634y^ receptor. The selected D4 aptamer inhibits neurite outgrowth and reverts the neoplastic phenotype of NIH/MEN2A cells *in vitro* [89] and in three dimensional collagen gel matrix cultures [90]. The HER-3 aptamer, a 2’-FY aptamer, known as A30, was isolated against the HER-3 monomeric extracellular domain; this prevents HER-2 signaling via HER-3 heterodimerization in cell culture [91]. 

Cell adhesion misregulation involved in metastasis has also been prevented by aptamers. The plasminogen activator inhibitor-1 (PAI-1) is overexpressed in breast cancer cells and binds to vitronectin, leading to the loss of adhesion. A 2’-FY aptamer known as SM-30, specific for plasminogen activator inhibitor-1 (PAI-1), restores cell adhesion to vitronectin-coated plates *in vitro* [92,93]. 

Another type of therapeutic signaling modulation is the targeting of nuclear factor *κ*B (NF-*κ*B) inside cells. Maher’s group isolated two RNA aptamers, α-p50 and R1, by two independent *in vitro* selection procedures. These bind NF-*κ*B p50 and p65 isoforms *in vitro* respectively [94,95]. These aptamers underwent further *ex vivo* selection using the yeast three-hybrid system [96,97]. The inhibition of NF-κB might be of therapeutic interest in different types of cancer, HIV-1 infection and inflammatory diseases. In fact, gene therapy with different anti-NF-κB aptamers administered via an adenoviral vector suppresses doxorubicin resistance *in vivo* in a lung tumor xenograft mouse model [98,99]. The inhibition of nucleophosmin oligomerization by an aptamer promotes higher p53 levels and, consistently, sensitizes cells to DNA-damaging-agent-induced apoptosis in cell culture [100].

The stimulation of the immune system can also be used in anti-cancer therapy. The activation of CD8^+^ T cells within a tumor would promote its cytotoxic involution. In this respect, the modulation of cytotoxic T-cell antigen-4 (CTLA-4), 4-1BB and OX40 receptors by aptamers has been explored. Multimeric aptamer forms frequently improve aptamer signaling properties and have proven especially importance in this area. It has been shown that an antagonist RNA aptamer against CTLA-4, a negative regulator of T-cell activation, inhibits its function [101], while an agonistic aptamer against 4-1BB, a major co-stimulatory receptor, leads to the activation of T cells [102]. Immunity against the tumor is induced *in vivo* in both cases. A specific aptamer for the dimeric murine OX40 combined with a dendritic cell-based tumor vaccine promotes tumor immunity in a xenograft melanoma model in mice [103]. 

Aptamers have also been shown able to promote tumor cell death. When expressed in cells, an aptamer selected against nucleophosmin was shown to prevent the latter’s oligomerization. Higher p53 levels were therefore promoted that led to apoptosis. In addition, the sensitivity of cells to DNA-damaging agents in cell culture was increased [100]. 

Research into anti-angiogenesis aptamers has provided some very interesting results. Sullenger’s group reported the selection of specific RNA aptamers against the *Ang1* and *Ang2* genes [36,104]. Aptamer ANG9-4 binds to *Ang1* and inhibits its signaling pathway, leading to the reduced survival of HUVEC cells *in vitro* [36]. Similarly, intraocularly administered aptamer 11-1.41 binds to *Ang2* and inhibits angiogenesis in rat corneal micropockets [104]. A 3’ deoxythymidine cap protects this aptamer from RNases. The PEGylated version of this molecule was shown to inhibit tumor angiogenesis and growth in an *in vivo* murine metastatic colon cancer model following systemic administration [105].

While the large majority of aptamers have been isolated by SELEX [106], the anti-proliferative DNA aptamer AS-1411 was developed based on observations that guanosine-rich oligonucleotides have antiproliferative effects in tumor cells [107]. Molecular studies have shown that this aptamer binds to the cell surface protein nucleolin and inhibits the activity of NF-KB, a ubiquitous transcription factor, through intracellular complex formation [108]. Clinical studies of AS-1411 have focused on patients with renal, pancreatic and other solid tumors. The aptamer was administered to patients as a continuous infusion for 4 or 7 days. 

Selectins are a family of cell adhesion molecules expressed by leukocytes, endothelial cells and platelets [109]. They are involved in a number of inflammatory diseases as well as tissue injury and infection. DNA selection against the L-selectin/IgG fusion protein (LS-Rg) was performed to find aptamers that could be tested *in vivo* [110]. Aptamers LD201, LD174 and LD196 all bound with a Kd of 1.8 nM at 37 ºC. Truncated versions of these aptamers inhibited SL-Rg binding to its ligand sialyl Lewis X (sLeX) with an IC_50_ of 3 nM. Aptamer LD201t1 blocked L-selectin-mediated adhesion of human lymphocytes and neutrophils and inhibited human cell trafficking to peripheral and mesenteric lymph nodes in SCID mice.

Platelet-derived growth factor (PDGF) is a ubiquitous mitogen and chemotactic growth factor in the form of three disulphide-linked dimers made of two homologous chains, A and B. It is involved in wound healing and is linked to the progression of numerous diseases, including atherosclerosis and glomerulonephritis [111,112]. A hallmark of malignant transformation is the loss of dependence on exogenous mitogenic stimulation; many tumor cell lines are thought to produce and secrete PDGF for this reason [113]. DNA selection against recombinant human PDGF-AB yielded DNA specific aptamers of the PDGF B-chain that bound with subnanomolar affinity [114]. The consensus secondary structure motif for most of the high-affinity ligands is a three-way helix junction with a three-nucleotide loop at the branch point. The PDGF aptamers inhibited the mitogenic effects of PDGF-BB in cells that expressed PDGF *β* receptors [114,115]. PEG-modified aptamers in a rat model of mesangioproliferative glomerulonephritis led to a 64% reduction in mitoses by day 6, and 78% by day 9. There was also a 95% reduction of proliferating mesangial cells by day 9 and a markedly reduced glomerular expression of the endogenous PDGF B-chain. Aptamer-treated animals also showed a reduced influx of monocytes/macrophages and the overproduction of glomerular extracellular matrix on day 6 [109]. Further studies revealed the inhibition of other disease mechanisms by the above aptamer in experimental glomerulonephritis [116,117]. 

The expression of PDGF *β* receptors in tumors is associated with increased interstitial fluid pressure (IFP) in the dermis. This reduces the gradient between capillaries and the interstitium and impedes the exchange of solutes, such as anticancer agents, over the capillary membrane [118]. Increasing this gradient may facilitate the transport of anticancer drugs to tumors [119]. To reduce the IFP, the PDGF-B aptamers [109] were tested in a rat tumor model. The treated animals had an IFP of 9.7 mm Hg compared to 14.6 mm Hg in scrambled-RNA-treated animals [120]. Another DNA aptamer known as E1-0030 that targets the PDGF-B subtype is currently undergoing Phase I clinical trials as an anti-vascular endothelial growth factor compound [121]. 

### 6.5. Anti-vascular diseases aptamers

Blood fluidity and blood vessel resistance are involved in numerous vascular diseases such as coronary and thrombotic syndromes and myocardial infarction. Certainly, these factors must be carefully controlled during coronary surgery. The proliferation of cardiac and vascular cells is key in the development of vessel resistance in diseases such as cardiac intimal hyperplasia, cardiac hypertrophy and atherosclerosis, as well as in the development of malignancies [122,123]. A recent study has reported the development of an RNA aptamer able to specifically recognize members of the E2F transcription factors involved in cell proliferation. The binding of the 2’-FY aptamer 8-2 mainly to E2F3, reduces intimal hyperplasia and the pathological proliferation and migration of vascular smooth muscle cells (VSMCs) after bypass surgery in a mouse model [124]. This aptamer avoids the side effects derived from cross reactivity with other members of the E2F family. 

In a different approach, SELEX has been performed with the E2F1 protein to find *in vitro* selected RNA aptamers that bind to and inhibit E2F activity. Clone E1 RNA was found to bind to E2F1 and blocked the latter’s attachment to its DNA binding site [125]. By impeding E2F activity, the E2F RNA aptamer inhibited S-phase induction by 90% compared to controls. Thus, both natural and *in vitro* selected aptamers appear able to limit cell proliferation.

**Figure 4:**
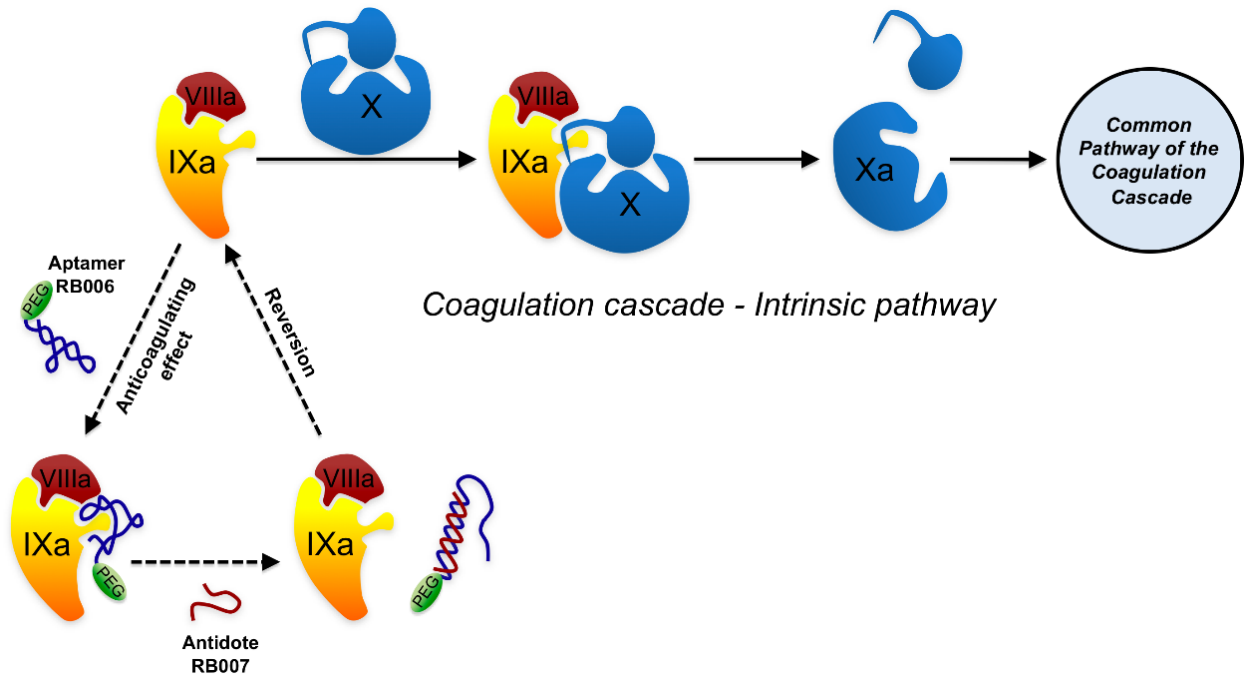
Mechanism of the apatamer-antidote pair for anticoagulant therapy.The intrinsic pathway of the blood coagulation cascade involves the activation of factor X. Anticoagulation system REG1 consists of RB006 (drug), an injectable RNA aptamer that specifically binds to activated factor IX (IXa) and prevents the proteolytic cleavage of factor X; and RB007 (antidote), a RNA antisense oligonucleotide that neutralizes the anticoagulating effect of the aptamer RB006. In the presence of the antidote, the aptamer is released from factor IXa and clotting parameters return to normal. Together with activated factor VIII (VIIIa), factor IXa catalyzes the cleavage of factor X (pro-enzyme) to yield activated factor X (Xa), which is required for the blood clotting cascade.

The coagulation signaling cascade offers several targets for the modulation of blood fluidity. The conversion of pro-thrombin to thrombin is delayed by a long half-life (15 h) 2’-FY aptamer targeting the catalyst factor VIIa in a dose dependent manner [126]. Nevertheless, the rapid restoration of cascade integrity is needed to prevent the harmful effects of coagulation deficiency. With this aim, an RNA-based aptamer-antidote system has been developed [127,128]. The REG-1 RNA aptamer targets factor XIa. An antisense RNA molecule was designed to specifically bind the 5’-half of the aptamer (the antidote). Binding of the antidote abolishes the aptamer’s binding to its target, thereby reversing the anticoagulant effect (Figure 4). Neither antidote nor aptamer have been seen to cause any adverse effect in Phase I clinical trials (Ia and Ib) when given to healthy people and patients with stable cardiovascular disease receiving antiplatelet therapy [61,63]. The results of another Phase I clinical trial (Ic) indicate that the anticoagulation effect can be modulated by varying the dose of antidote RNA [62]. In a recent Phase IIa trial, percutaneous coronary injection of the aptamer increased the activated clotting time (ACT) in patients immediately after its administration, reaching values close to those obtained with heparin. ACT values were restored 15 min after the administration of the antidote [129]. 

Thrombin is a natural target for anticoagulation therapy and numerous aptamers have been generated with different capacities to inhibit its activity *in vitro* [56,70]. The Archemix Corp., in collaboration with ARCA Biopharma Inc. (formerly Nuvelo Inc.), has tested NU-172, a DNA aptamer against thrombin. In Phase I clinical trials, NU-172 was administered intravenously as a continuous infusion to healthy volunteers. The results showed an increase in activated clotting time with a return to baseline when administration ceased (see Archemix Corp. website). A Phase II clinical study is currently underway with the goal of using NU-172 in coronary artery bypass graft surgery and percutaneous coronary intervention. 

A DNA/RNA aptamer conjugated to PEG, known as ARC-1779, generated against vWF (von Willebrand factor), a central factor in the adhesion of platelets to the endothelial surface at vascular injury sites [130], has been examined in a Phase I trial in healthy volunteers. The aptamer increased platelet function in a whole-blood assay sensitive to vWF-mediated platelet inhibition. Moreover, a slow intravenous bolus followed by 4 h of continuous infusion inhibited more than 95% of vWF function, which returned to baseline over 12-16 h after administration was suspended.

NF-*κ*B is involved in inflammation responses and modulates the synthesis of chemokines, interferons, major histocompatibility complex (MHC) proteins, growth factors, and the cell adhesion molecules that play a role in ischemia-reperfusion injuries seen in most myocardial infarctions [131]. A natural double stranded DNA aptamer was found that binds to NF-*κ*B with high affinity. In a rat cardiac ischemia-reperfusion model, this aptamer significantly reduced the expected injury [115]. In a rat cardioplegic arrest model, animals transfected with the NF-*κ*B DNA aptamer showed improved recovery of left ventricular function as well as coronary flow three days post-transfection compared to scrambled-DNA controls (97% *vs.* 61%) [132]. The aptamer-treated group also showed a lower percentage of neutrophil adhesion to endothelial cells (38% *vs.* 81%) and a lower level of interleukin IL-8 (109 *vs.* 210 ng/mg). The same aptamer was also studied in a murine model of nephritis, in which it abolished glomerular inflammation and the expression of inflammatory markers IL-1*α*, IL-1*β*, IL-6, ICAM-2 (intracellular adhesion molecule 2), and VCAM-1 (vascular cell adhesion molecule 1) [133]. A rat global brain ischemia model showed inhibition of TNF-*α*, IL-1*β* and ICAM-1 expression in NF-*κ*B aptamer-treated animals after 1 h of ischemia. Moreover, 7 days after ischemia, neuronal damage was significantly attenuated in the NF-*κ*B-aptamer-treated group compared to controls [134].

### 6.6. Anti-pathogen applications of selection procedures.

The proteins of different pathogens have also attracted the attention of researchers as targets for inhibitory nucleic acids. A recent study reported the use of a 2’-FY aptamer against the extracellular domain of the erythrocyte membrane protein 1 (PfEMP1) of the parasitic protozoan *Plasmodium falciparum*, achieving the efficient inhibition of erythrocyte rosseting in blood cultures [135].

RNA viruses, especially HIV-1 and HCV, have been the main targets for therapeutic nucleic acids with catalytic activity. Rossi’s group designed a very interesting *ex vivo* selection procedure to identify anti-HIV hammerhead ribozymes. A pool of hammerhead catalytic domains containing randomized binding arm sequences was assayed against an HIV-1 chimera containing the thymidine kinase gene. After cell transfection of the ribozyme population expressed under the control of the U16snoRNA promoter, gancyclovir-resistant cells were selected. Such antibiotic resistance suggests the presence of an active anti-HIV ribozyme [136].

Banerjea’s group designed a strategy to identify accessible cleavage sites within the HIV-1 gag RNA and to pick out DNAzymes effective against this target [137]. Two DNAzyme variant populations were synthesized. The specificity of the first was limited to all possible AUGs in the target RNA, whereas the second population was designed to cleave any potential target site. These options were made possible by either fixing the nucleotides immediately preceding the catalytic motif to CA (for AUG cleavage) or totally randomizing the seven bases on either side of the catalytic motif. DNAzymes selected from both populations showed target-specific cleavage activities in the presence of Mg^2+^, and significantly interfered with HIV-1-specific gene expression. This strategy could be used for the selection of effective target sites in any target RNA [137].

Viral proteins are favorite targets for the development of therapeutic aptamers. RNA aptamers have been selected against different viral enzymes and proteins involved in host-cell interactions, such as HIV-1 reverse transcriptase (RT), HIV-1 glycoprotein 120, HCV RNA-dependent RNA polymerase (RdRp), SARS coronavirus NTPase/helicase, and the hemagglutinin protein of human influenza virus B, all of which show efficient viral inhibition [138,139,140,141,142,143]. Human influenza B virus hemagglutinin protein has been inhibited *in vitro* by the RNA pool resulting from 15 rounds of *in vitro* selection [143]. Further studies are required for the identification of independent aptamers.

Recently, a DNA aptamer (termed 93del) has been described that adopts a novel type of dimeric quadruplex folding and shows anti-HIV1 integrase activity in the nanomolar range *in vitro* by blocking several catalytic amino acid residues essential for integrase function. Several other G-rich DNA aptamers have been identified as remarkable HIV1-IN inhibitors with IC50 values in the nanomolar range. T30695 is one such aptamer, and has been well studied in recent years, [144,145]. 

Astier-Gi’s group described the characterization of two DNA aptamers (27v and 127v) that specifically bind to hepatitis C virus (HCV) RNA polymerase (NS5B), inhibiting its activity *in vitro* [146]. Aptamer 27v competed with the RNA template for binding to the enzyme and blocked both the initiation and elongation phases of RNA synthesis, whereas aptamer 127v exclusively inhibited initiation events. The authors also determined that, in addition to an *in vitro* inhibitory effect on RNA synthesis, aptamer 27v interfered with the multiplication of HCV JFH1 in Huh7 cells. The efficient cellular entry of these short DNAs, and the inhibitory effect observed in human cells infected with HCV, indicate that aptamers are useful tools for the study of HCV RNA synthesis; their therapeutic against HCV infection is also attractive [146].

An alternative and innovative potential therapeutic approach that has attracted much hope is the targeting of the structural domains of viral genomes which are frequently concentrated within untranslated terminal regions (UTRs). The HIV-1 trans-activation response (TAR) element is a polyfunctional RNA domain mainly involved in the activation of transcription by its binding to the viral protein Tat. Aptamer R06 binds the TAR element *in vitro* by a loop-loop interaction [147]. The intracellular expression of the aptamer by the nucleolar U16 RNA promoter inhibits HIV-1 infection by more than 90%. This strategy takes advantage of HIV-1 RNA nucleolar-trafficking to efficiently colocalize the aptamer and target [148]. The same R06 aptamer has been improved by the inclusion of extra binding RNA domains targeting additional 5’-UTR sites [149]. Our own results show that pre-synthesized aptamers, or aptamers produced intracellularly via U6 RNA promoter-driven expression, both with multiple binding sites targeting the whole HIV-1 5’-UTR, efficiently inhibit HIV-1 replication in cell culture (Sánchez-Luque *et al*., unpublished). 

There have been different attempts to isolate RNA aptamers against different domains of the HCV internal ribosome entry site (IRES) located within the 5’-UTR [150,151,152]. Aptamer 3-07 binds domain III-IV of HCV IRES inhibiting its activity in cell-free systems [151]. This inhibition was further improved by conjugation of 3-07 with 2-02 in a chimeric molecule that targets domain II [153]. RNA aptamer AP30 isolated against the HCV (-) strand IRES domain I partially inhibits HCV RNA replicase-mediated (+) strand synthesis, probably by interfering with (-) strand binding [154]. A step forward was the design of an innovative selection approach to identify chimeric ribozyme/aptamer RNA molecules against the entire HCV-IRES. Each selection cycle includes two consecutive selection steps for binding and cleavage of the viral RNA [155]. After six selection rounds, seven families of inhibitor RNAs were identified simultaneously targeting two sites within the HCV-IRES, one for each inhibitory domain. These chimeric RNA inhibitors promoted IRES inhibition by up to 95% in cell extracts, identifying new targets for the development of anti-HCV agents. Further characterization revealed up to 50% inhibition of viral translation and replication in a human liver cell line [156,157].

### 6.7. Aptamers working as antidotes.

The ability of SELEX to generate aptamers against any kind of target provides the possibility of their being used as therapeutic antidotes or palliatives. Ricin is a toxic heterodimeric lectin from the castor bean plant *Ricinus communis*. It disrupts protein synthesis by inactivation of the ribosomes. An aptamer isolated against the A chain partially restores protein synthesis levels in cell free translation systems and cell cultures [158]. A different approach has been explored for cocaine and anti-convulsant MK-801 alleviation. Both compounds preferentially bind the open isoform of nicotine acetylcholine receptors (nAChRs) found at neuromuscular junctions, autonomic ganglia and in the central nervous system. RNA aptamers developed against nAChRs with equal binding affinity for both open and closed sodium-potasium channel isoforms partially restore isoform equilibrium, alleviating drug channel inhibition in cells [159,160,161].

### 6.8. Aptamers as delivery tools

Aptamers can distinguish between different isoforms of the same protein or different members of the same family. Aptamer-siRNA/toxin conjugates have been developed to deliver therapeutic agents within a specific target cell. *Ex vivo* selection procedures performed against a specific cell type have yielded aptamers that are very efficiently taken up by those cells; they could therefore be used as a specific delivery tool. The TTA1 aptamer of Tenascin C, selected against the human glioma U251 cell line [110,162], was efficiently taken up by human tumor cells in a xenograft glioblastoma and breast tumor model [163]. Aptamers targeting the prostate-specific membrane antigen (PSMA) are those most used as delivery tools. Coffey’s group reported two 2’-FY RNA aptamers, A9 and A10, that bind specifically recombinant PSMA [164]. Since PSMA is continually recycled from the cell surface, these aptamers are appropriate vehicles for delivering therapeutic compounds via the endosomal pathway. Small interfering RNAs against pro-survival factors over-expressed in prostate cancer cells, such as Plk-1 and Bcl-2 and eukaryotic elongation factor 2, have been delivered by the A10 aptamer [165] [166]. The aptamer-driven uptake of Plk-1 Bcl-2 siRNAs leads to the death of PSMA-positive cells and tumor regression following intra-tumoral injection in mice xenograft models [165]. The improvement of the delivery vehicles were achieved in several ways and tumor regression through the almost complete loss of cell viability was observed when the eukaryotic elongation factor 2 siRNA was delivered by a dimeric aptamer [166].

The delivery of pharmacological compounds by aptamers has also been studied by different approaches. Doxorubicin (Dox) is a chemotherapeutic intercalating agent used against cancer. Dox intercalates between the aromatic rings of the GC pairs of the A10 aptamer, enhancing its cytotoxicity against PSMA-expressing cells and reducing the same in non-expressing cells [167]. The same results have been reported when using the A9-genolin conjugate, a toxin that blocks protein synthesis [168]. Side toxicity of the chemotherapeutic compound can be further prevented by encapsulating it within nanoparticles or nanoconjugates [169,170,171].

Aptamer B40, specific for HIV-1 gp120 [140], has also been used to deliver siRNA against infected cells. HIV-1 gp120 is found on the surface of infected cells and promotes cell fusion. Consequently, besides neutralizing infective particles, the B40 aptamer can be used for anti-HIV-1 siRNA delivery to infected cells. The first attempt involved a chimera of the B40 aptamer and siRNA targeting the overlapping *revtat* region [172]. The chimera was internalized in an aptamer-dependent manner, with inhibition dependent on the interference machinery. The aptamer-siRNA linkage was further improved for the easy combination of siRNAs to the aptamer and better siRNA processing by the cellular machinery. This improved chimera resulted in the specific inhibition of HIV-1 replication and infectivity in PBMC and cultured CEM T cells [173]. 

Finally, aptamers can also be used to colocalize RNA inhibitors with their specific target molecules at the subcellular level. Aptamers can target RNA domains, e.g., in viral RNA genomes; they can therefore improve trans-cleaving ribozymes by anchoring them to their target. Chimeric molecules composed of a hammerhead ribozyme and an aptamer both targeting the HCV IRES have efficiently inhibited IRES activity in human hepatocyte cell cultures [156,157].

## 7. Conclusions

The procedures used to identify DNA and RNA molecules of interest in large populations of variant nucleic acid molecules have contributed significantly to the development of nucleic acid-based therapeutic drugs. Aptamers show high specificity for their targets and have low toxicity and immunogenicity profiles. Since the 1990s, the design and isolation of specific aptamers using selection and evolution techniques has been optimized and even automated. This has lead to great advances in our knowledge of aptamers as therapeutic agents and has expanded our bank of inhibitory nucleic acids and their possible targets, which now include cytokines, cell receptors, viruses and even whole cells. An aptamer drug is now on the market, and several mid and late clinical trials in progress that appears to confirm the great potential of these tools. With the improvement and optimization of selection strategies, and the ongoing discoveries made in this field, success in the development of nucleic acid-based therapeutics protocols might be predicted.

## References

[1] Mills D.R., Peterson R.L., Spiegelman S. (1967). An extracellular Darwinian experiment with a self-duplicating nucleic acid molecule. Proc. Natl. Acad. Sci. USA.

[2] Green R., Ellington A.D., Szostak J.W. (1990). *In vitro* genetic analysis of the Tetrahymena self-splicing intron. Nature.

[3] Ellington A.D., Szostak J.W. (1990). *In vitro* selection of RNA molecules that bind specific ligands. Nature.

[4] Robertson D.L., Joyce G.F. (1990). Selection *in vitro* of an RNA enzyme that specifically cleaves single-stranded DNA. Nature.

[5] Tuerk C., Gold L. (1990). Systematic evolution of ligands by exponential enrichment: RNA ligands to bacteriophage T4 DNA polymerase. Science.

[6] Burke J.M., Berzal-Herranz A. (1993). *In vitro* selection and evolution of RNA: applications for catalytic RNA, molecular recognition, and drug discovery. FASEB J..

[7] Berzal-Herranz A. (1996). *In vitro* selection of hairpin ribozymes. J. Hepatol..

[8] Breaker R.R. (1997). *In Vitro* Selection of Catalytic Polynucleotides. Chem. Rev..

[9] Wilson D.S., Szostak J.W. (1999). *In vitro* selection of functional nucleic acids. Annu. Rev. Biochem..

[10] Nieuwlandt D. (2000). *In vitro* selection of functional nucleic acid sequences. Curr. Issues Mol. Biol..

[11] Dausse E., Cazenave C., Rayner B., Toulme J.J. (2005). *In vitro* selection procedures for identifying DNA and RNA aptamers targeted to nucleic acids and proteins. Methods Mol. Biol..

[12] Romero-López C., Díaz-González R., Berzal-Herranz A. (2007). RNA selection and evolution *in vitro*: powerful techniques for the analysis and identification of new molecular tools. Biotechnol. Biotechnol. Equip..

[13] Dausse E., Da Rocha Gomes S., Toulme J.J. (2009). Aptamers: a new class of oligonucleotides in the drug discovery pipeline?. Curr. Opin. Pharmacol..

[14] Gevertz J., Gan H.H., Schlick T. (2005). *In vitro* RNA random pools are not structurally diverse: a computational analysis. RNA.

[15] Jarosch F., Buchner K., Klussmann S. (2006). *In vitro* selection using a dual RNA library that allows primerless selection. Nucleic Acids Res..

[16] Vater A., Jarosch F., Buchner K., Klussmann S. (2003). Short bioactive Spiegelmers to migraine-associated calcitonin gene-related peptide rapidly identified by a novel approach: tailored-SELEX. Nucleic Acids Res..

[17] Pan W., Xin P., Clawson G.A. (2008). Minimal primer and primer-free SELEX protocols for selection of aptamers from random DNA libraries. Biotechniques.

[18] Berzal-Herranz A., Joseph S., Burke J.M. (1992). *In vitro* selection of active hairpin ribozymes by sequential RNA-catalyzed cleavage and ligation reactions. Genes Dev..

[19] Chen X., Li N., Ellington A.D. (2007). Ribozyme catalysis of metabolism in the RNA world. Chem. Biodivers..

[20] Fitzwater T., Polisky B. (1996). A SELEX primer. Methods Enzymol..

[21] Naimuddin M., Kitamura K., Kinoshita Y., Honda-Takahashi Y., Murakami M., Ito M., Yamamoto K., Hanada K., Husimi Y., Nishigaki K. (2007). Selection-by-function: efficient enrichment of cathepsin E inhibitors from a DNA library. J. Mol. Recognit..

[22] Wu L., Curran J.F. (1999). An allosteric synthetic DNA. Nucleic Acids Res..

[23] Buskirk A.R., Landrigan A., Liu D.R. (2004). Engineering a ligand-dependent RNA transcriptional activator. Chem. Biol..

[24] Buskirk A.R., Kehayova P.D., Landrigan A., Liu D.R. (2003). *In vivo* evolution of an RNA-based transcriptional activator. Chem. Biol..

[25] Wieland M., Berschneider B., Erlacher M.D., Hartig J.S. (2010). Aptazyme-mediated regulation of 16S ribosomal RNA. Chem. Biol..

[26] Nomura Y., Yokobayashi Y. (2007). Reengineering a natural riboswitch by dual genetic selection. J. Am. Chem. Soc..

[27] Chen X., Denison L., Levy M., Ellington A.D. (2009). Direct selection for ribozyme cleavage activity in cells. RNA.

[28] Behlke M.A. (2008). Chemical modification of siRNAs for *in vivo* use. Oligonucleotides.

[29] Thiel K.W., Giangrande P.H. (2009). Therapeutic applications of DNA and RNA aptamers. Oligonucleotides.

[30] Singh Y., Murat P., Defrancq E. Recent developments in oligonucleotide conjugation. Chem. Soc. Rev..

[31] Said Hassane F., Saleh A.F., Abes R., Gait M.J., Lebleu B. (2010). Cell penetrating peptides: overview and applications to the delivery of oligonucleotides. Cell Mol. Life Sci..

[32] Degols G., Leonetti J.P., Lebleu B. (1992). Sequence-specific activity of antisense oligonucleotides conjugated to poly (L-lysine) carriers. Ann. N.Y. Acad. Sci..

[33] Lundquist J.J., Toone E.J. (2002). The cluster glycoside effect. Chem. Rev..

[34] Gissot A., Camplo M., Grinstaff M.W., Barthelemy P. (2008). Nucleoside, nucleotide and oligonucleotide based amphiphiles: a successful marriage of nucleic acids with lipids. Org. Biomol. Chem..

[35] Proske D., Gilch S., Wopfner F., Schatzl H.M., Winnacker E.L., Famulok M. (2002). Prion-protein-specific aptamer reduces PrPSc formation. Chembiochem.

[36] White R.R., Roy J.A., Viles K.D., Sullenger B.A., Kontos C.D. (2008). A nuclease-resistant RNA aptamer specifically inhibits angiopoietin-1-mediated Tie2 activation and function. Angiogenesis.

[37] Kumar R., Singh S.K., Koshkin A.A., Rajwanshi V.K., Meldgaard M., Wengel J. (1998). The first analogues of LNA (locked nucleic acids): phosphorothioate-LNA and 2'-thio-LNA. Bioorg. Med. Chem. Lett..

[38] Bondensgaard K., Petersen M., Singh S.K., Rajwanshi V.K., Kumar R., Wengel J., Jacobsen J.P. (2000). Structural studies of LNA:RNA duplexes by NMR: conformations and implications for RNase H activity. Chemistry.

[39] Petersen M., Nielsen C.B., Nielsen K.E., Jensen G.A., Bondensgaard K., Singh S.K., Rajwanshi V.K., Koshkin A.A., Dahl B.M., Wengel J., Jacobsen J.P. (2000). The conformations of locked nucleic acids (LNA). J. Mol. Recognit..

[40] Veedu R.N., Wengel J. (2010). Locked nucleic acids: promising nucleic acid analogs for therapeutic applications. Chem. Biodivers..

[41] Veedu R.N., Vester B., Wengel J. (2007). *In vitro* incorporation of LNA nucleotides. Nucleosides Nucleotides Nucleic Acids.

[42] Veedu R.N., Vester B., Wengel J. (2008). Polymerase chain reaction and transcription using locked nucleic acid nucleotide triphosphates. J. Am. Chem. Soc..

[43] Veedu R.N., Vester B., Wengel J. (2009). Efficient enzymatic synthesis of LNA-modified DNA duplexes using KOD DNA polymerase. Org. Biomol. Chem..

[44] Klussmann S., Nolte A., Bald R., Erdmann V.A., Furste J.P. (1996). Mirror-image RNA that binds D-adenosine. Nat. Biotechnol..

[45] Nolte A., Klussmann S., Bald R., Erdmann V.A., Furste J.P. (1996). Mirror-design of L-oligonucleotide ligands binding to L-arginine. Nat. Biotechnol..

[46] Wlotzka B., Leva S., Eschgfaller B., Burmeister J., Kleinjung F., Kaduk C., Muhn P., Hess-Stumpp H., Klussmann S. (2002). *In vivo* properties of an anti-GnRH Spiegelmer: an example of an oligonucleotide-based therapeutic substance class. Proc. Natl. Acad. Sci. USA.

[47] Helmling S., Maasch C., Eulberg D., Buchner K., Schroder W., Lange C., Vonhoff S., Wlotzka B., Tschop M.H., Rosewicz S., Klussmann S. (2004). Inhibition of ghrelin action *in vitro* and *in vivo* by an RNA-Spiegelmer. Proc. Natl. Acad. Sci. USA.

[48] Santoro S.W., Joyce G.F. (1997). A general purpose RNA-cleaving DNA enzyme. Proc. Natl. Acad. Sci. USA.

[49] Doherty E.A., Doudna J.A. (2000). Ribozyme structures and mechanisms. Annu. Rev. Biochem..

[50] Schubert S., Furste J.P., Werk D., Grunert H.P., Zeichhardt H., Erdmann V.A., Kurreck J. (2004). Gaining target access for deoxyribozymes. J. Mol. Biol..

[51] Isaka Y. (2007). DNAzymes as potential therapeutic molecules. Curr. Opin. Mol. Ther..

[52] Reyes-Darias J.A., Sanchez-Luque F.J., Berzal-Herranz A. (2008). Inhibition of HIV-1 replication by RNA-based strategies. Curr. HIV Res..

[53] Cullen B.R., Greene W.C. (1989). Regulatory pathways governing HIV-1 replication. Cell.

[54] Marciniak R.A., Garcia-Blanco M.A., Sharp P.A. (1990). Identification and characterization of a HeLa nuclear protein that specifically binds to the trans-activation-response (TAR) element of human immunodeficiency virus. Proc. Natl. Acad. Sci. USA.

[55] Sullenger B.A., Gallardo H.F., Ungers G.E., Gilboa E. (1990). Overexpression of TAR sequences renders cells resistant to human immunodeficiency virus replication. Cell.

[56] White R., Rusconi C., Scardino E., Wolberg A., Lawson J., Hoffman M., Sullenger B. (2001). Generation of species cross-reactive aptamers using "toggle" SELEX. Mol. Ther..

[57] Ng E.W., Shima D.T., Calias P., Cunningham E.T., Guyer D.R., Adamis A.P. (2006). Pegaptanib, a targeted anti-VEGF aptamer for ocular vascular disease. Nat. Rev. Drug Discov..

[58] Ciulla T.A., Rosenfeld P.J. (2009). Antivascular endothelial growth factor therapy for neovascular age-related macular degeneration. Curr. Opin. Ophthalmol..

[59] Diener J.L., Daniel Lagasse H.A., Duerschmied D., Merhi Y., Tanguay J.F., Hutabarat R., Gilbert J., Wagner D.D., Schaub R. (2009). Inhibition of von Willebrand factor-mediated platelet activation and thrombosis by the anti-von Willebrand factor A1-domain aptamer ARC1779. J. Thromb. Haemost..

[60] Gilbert J.C., DeFeo-Fraulini T., Hutabarat R.M., Horvath C.J., Merlino P.G., Marsh H.N., Healy J.M., Boufakhreddine S., Holohan T.V., Schaub R.G. (2007). First-in-human evaluation of anti von Willebrand factor therapeutic aptamer ARC1779 in healthy volunteers. Circulation.

[61] Dyke C.K., Steinhubl S.R., Kleiman N.S., Cannon R.O., Aberle L.G., Lin M., Myles S.K., Melloni C., Harrington R.A., Alexander J.H., Becker R.C., Rusconi C.P. (2006). First-in-human experience of an antidote-controlled anticoagulant using RNA aptamer technology: a phase 1a pharmacodynamic evaluation of a drug-antidote pair for the controlled regulation of factor IXa activity. Circulation.

[62] Chan M.Y., Rusconi C.P., Alexander J.H., Tonkens R.M., Harrington R.A., Becker R.C. (2008). A randomized, repeat-dose, pharmacodynamic and safety study of an antidote-controlled factor IXa inhibitor. J. Thromb. Haemost..

[63] Chan M.Y., Cohen M.G., Dyke C.K., Myles S.K., Aberle L.G., Lin M., Walder J., Steinhubl S.R., Gilchrist I.C., Kleiman N.S., Vorchheimer D.A., Chronos N., Melloni C., Alexander J.H., Harrington R.A., Tonkens R.M., Becker R.C., Rusconi C.P. (2008). Phase 1b randomized study of antidote-controlled modulation of factor IXa activity in patients with stable coronary artery disease. Circulation.

[64] Bates P.J., Laber D.A., Miller D.M., Thomas S.D., Trent J.O. (2009). Discovery and development of the G-rich oligonucleotide AS1411 as a novel treatment for cancer. Exp. Mol. Pathol..

[65] Soundararajan S., Chen W., Spicer E.K., Courtenay-Luck N., Fernandes D.J. (2008). The nucleolin targeting aptamer AS1411 destabilizes Bcl-2 messenger RNA in human breast cancer cells. Cancer Res..

[66] Ireson C.R., Kelland L.R. (2006). Discovery and development of anticancer aptamers. Mol. Cancer Ther..

[67] Jo N., Mailhos C., Ju M., Cheung E., Bradley J., Nishijima K., Robinson G.S., Adamis A.P., Shima D.T. (2006). Inhibition of platelet-derived growth factor B signaling enhances the efficacy of anti-vascular endothelial growth factor therapy in multiple models of ocular neovascularization. Am. J. Pathol..

[68] Sennino B., Falcon B.L., McCauley D., Le T., McCauley T., Kurz J.C., Haskell A., Epstein D.M., McDonald D.M. (2007). Sequential loss of tumor vessel pericytes and endothelial cells after inhibition of platelet-derived growth factor B by selective aptamer AX102. Cancer Res..

[69] Biesecker G., Dihel L., Enney K., Bendele R.A. (1999). Derivation of RNA aptamer inhibitors of human complement C5. Immunopharmacology.

[70] Bock L.C., Griffin L.C., Latham J.A., Vermaas E.H., Toole J.J. (1992). Selection of single-stranded DNA molecules that bind and inhibit human thrombin. Nature.

[71] Griffin L.C., Tidmarsh G.F., Bock L.C., Toole J.J., Leung L.L. (1993). *In vivo* anticoagulant properties of a novel nucleotide-based thrombin inhibitor and demonstration of regional anticoagulation in extracorporeal circuits. Blood.

[72] DeAnda A., Coutre S.E., Moon M.R., Vial C.M., Griffin L.C., Law V.S., Komeda M., Leung L.L., Miller D.C. (1994). Pilot study of the efficacy of a thrombin inhibitor for use during cardiopulmonary bypass. Ann. Thorac. Surg..

[73] Shukla D., Namperumalsamy P., Goldbaum M., Cunningham E.T. (2007). Pegaptanib sodium for ocular vascular disease. Indian J. Ophthalmol..

[74] Ruckman J., Green L.S., Beeson J., Waugh S., Gillette W.L., Henninger D.D., Claesson-Welsh L., Janjic N. (1998). 2'-Fluoropyrimidine RNA-based aptamers to the 165-amino acid form of vascular endothelial growth factor (VEGF165). Inhibition of receptor binding and VEGF-induced vascular permeability through interactions requiring the exon 7-encoded domain. J. Biol. Chem..

[75] Ferrara N., Gerber H.P., LeCouter J. (2003). The biology of VEGF and its receptors. Nat. Med..

[76] Cunningham E.T., Adamis A.P., Altaweel M., Aiello L.P., Bressler N.M., D'Amico D.J., Goldbaum M., Guyer D.R., Katz B., Patel M., Schwartz S.D. (2005). A phase II randomized double-masked trial of pegaptanib, an anti-vascular endothelial growth factor aptamer, for diabetic macular edema. Ophthalmology.

[77] Lee K.S., Park S.J., Kim S.R., Min K.H., Lee K.Y., Choe Y.H., Hong S.H., Lee Y.R., Kim J.S., Hong S.J., Lee Y.C. (2008). Inhibition of VEGF blocks TGF-beta1 production through a PI3K/Akt signalling pathway. Eur. Respir. J..

[78] Huang Z., Pei W., Jayaseelan S., Shi H., Niu L. (2007). RNA aptamers selected against the GluR2 glutamate receptor channel. Biochemistry.

[79] Huang Z., Pei W., Han Y., Jayaseelan S., Shekhtman A., Shi H., Niu L. (2009). One RNA aptamer sequence, two structures: a collaborating pair that inhibits AMPA receptors. Nucleic Acids Res..

[80] Wang Y., Khaing Z.Z., Li N., Hall B., Schmidt C.E., Ellington A.D. (2010). Aptamer antagonists of myelin-derived inhibitors promote axon growth. PLoS One.

[81] Rhie A., Kirby L., Sayer N., Wellesley R., Disterer P., Sylvester I., Gill A., Hope J., James W., Tahiri-Alaoui A. (2003). Characterization of 2'-fluoro-RNA aptamers that bind preferentially to disease-associated conformations of prion protein and inhibit conversion. J. Biol. Chem..

[82] Doring G. (1994). The role of neutrophil elastase in chronic inflammation. Am. J. Respir. Crit. Care Med..

[83] Smith D., Kirschenheuter G.P., Charlton J., Guidot D.M., Repine J.E. (1995). *In vitro* selection of RNA-based irreversible inhibitors of human neutrophil elastase. Chem. Biol..

[84] Charlton J., Kirschenheuter G.P., Smith D. (1997). Highly potent irreversible inhibitors of neutrophil elastase generated by selection from a randomized DNA-valine phosphonate library. Biochemistry.

[85] Bless N.M., Smith D., Charlton J., Czermak B.J., Schmal H., Friedl H.P., Ward P.A. (1997). Protective effects of an aptamer inhibitor of neutrophil elastase in lung inflammatory injury. Curr. Biol..

[86] Gounni A.S., Lamkhioued B., Ochiai K., Tanaka Y., Delaporte E., Capron A., Kinet J.P., Capron M. (1994). High-affinity IgE receptor on eosinophils is involved in defence against parasites. Nature.

[87] Sutton B.J., Gould H.J. (1993). The human IgE network. Nature.

[88] Wiegand T.W., Williams P.B., Dreskin S.C., Jouvin M.H., Kinet J.P., Tasset D. (1996). High-affinity oligonucleotide ligands to human IgE inhibit binding to Fc epsilon receptor I. J. Immunol..

[89] Cerchia L., Duconge F., Pestourie C., Boulay J., Aissouni Y., Gombert K., Tavitian B., de Franciscis V., Libri D. (2005). Neutralizing aptamers from whole-cell SELEX inhibit the RET receptor tyrosine kinase. PLoS Biol..

[90] Vento M.T., Iuorio M., Netti P.A., Duconge F., Tavitian B., Franciscis V., Cerchia L. (2008). Distribution and bioactivity of the Ret-specific D4 aptamer in three-dimensional collagen gel cultures. Mol. Cancer Ther..

[91] Chen C.H., Chernis G.A., Hoang V.Q., Landgraf R. (2003). Inhibition of heregulin signaling by an aptamer that preferentially binds to the oligomeric form of human epidermal growth factor receptor-3. Proc. Natl. Acad. Sci. USA.

[92] Blake C.M., Sullenger B.A., Lawrence D.A., Fortenberry Y.M. (2009). Antimetastatic potential of PAI-1-specific RNA aptamers. Oligonucleotides.

[93] Madsen J.B., Dupont D.M., Andersen T.B., Nielsen A.F., Sang L., Brix D.M., Jensen J.K., Broos T., Hendrickx M.L., Christensen A.N., Kjems J., Andreasen P.A. (2010). RNA Aptamers as Conformational Probes and Regulatory Agents for Plasminogen Activator Inhibitor-1. Biochemistry.

[94] Lebruska L.L., Maher L.J. (1999). Selection and characterization of an RNA decoy for transcription factor NF-kappa B. Biochemistry.

[95] Wurster S.E., Maher L.J. (2008). Selection and characterization of anti-NF-kappaB p65 RNA aptamers. RNA.

[96] Cassiday L.A., Maher L.J. (2003). Yeast genetic selections to optimize RNA decoys for transcription factor NF-kappa B. Proc. Natl. Acad. Sci. USA.

[97] Wurster S.E., Bida J.P., Her Y.F., Maher L.J. (2009). Characterization of anti-NF-kappaB RNA aptamer-binding specificity *in vitro* and in the yeast three-hybrid system. Nucleic Acids Res..

[98] Mi J., Zhang X., Rabbani Z.N., Liu Y., Su Z., Vujaskovic Z., Kontos C.D., Sullenger B.A., Clary B.M. (2006). H1 RNA polymerase III promoter-driven expression of an RNA aptamer leads to high-level inhibition of intracellular protein activity. Nucleic Acids Res..

[99] Mi J., Zhang X., Rabbani Z.N., Liu Y., Reddy S.K., Su Z., Salahuddin F.K., Viles K., Giangrande P.H., Dewhirst M.W., Sullenger B.A., Kontos C.D., Clary B.M. (2008). RNA aptamer-targeted inhibition of NF-kappa B suppresses non-small cell lung cancer resistance to doxorubicin. Mol. Ther..

[100] Jian Y., Gao Z., Sun J., Shen Q., Feng F., Jing Y., Yang C. (2009). RNA aptamers interfering with nucleophosmin oligomerization induce apoptosis of cancer cells. Oncogene.

[101] Santulli-Marotto S., Nair S.K., Rusconi C., Sullenger B., Gilboa E. (2003). Multivalent RNA aptamers that inhibit CTLA-4 and enhance tumor immunity. Cancer Res..

[102] McNamara J.O., Kolonias D., Pastor F., Mittler R.S., Chen L., Giangrande P.H., Sullenger B., Gilboa E. (2008). Multivalent 4-1BB binding aptamers costimulate CD8+ T cells and inhibit tumor growth in mice. J. Clin. Invest..

[103] Dollins C.M., Nair S., Boczkowski D., Lee J., Layzer J.M., Gilboa E., Sullenger B.A. (2008). Assembling OX40 aptamers on a molecular scaffold to create a receptor-activating aptamer. Chem. Biol..

[104] White R.R., Shan S., Rusconi C.P., Shetty G., Dewhirst M.W., Kontos C.D., Sullenger B.A. (2003). Inhibition of rat corneal angiogenesis by a nuclease-resistant RNA aptamer specific for angiopoietin-2. Proc. Natl. Acad. Sci. USA.

[105] Sarraf-Yazdi S., Mi J., Moeller B.J., Niu X., White R.R., Kontos C.D., Sullenger B.A., Dewhirst M.W., Clary B.M. (2008). Inhibition of *in vivo* tumor angiogenesis and growth via systemic delivery of an angiopoietin 2-specific RNA aptamer. J. Surg. Res..

[106] Nimjee S.M., Rusconi C.P., Sullenger B.A. (2005). Aptamers: an emerging class of therapeutics. Annu. Rev. Med..

[107] Bates P.J., Kahlon J.B., Thomas S.D., Trent J.O., Miller D.M. (1999). Antiproliferative activity of G-rich oligonucleotides correlates with protein binding. J. Biol. Chem..

[108] Girvan A.C., Teng Y., Casson L.K., Thomas S.D., Juliger S., Ball M.W., Klein J.B., Pierce W.M., Barve S.S., Bates P.J. (2006). AGRO100 inhibits activation of nuclear factor-kappaB (NF-kappaB) by forming a complex with NF-kappaB essential modulator (NEMO) and nucleolin. Mol. Cancer Ther..

[109] Floege J., Ostendorf T., Janssen U., Burg M., Radeke H.H., Vargeese C., Gill S.C., Green L.S., Janjic N. (1999). Novel approach to specific growth factor inhibition in vivo: antagonism of platelet-derived growth factor in glomerulonephritis by aptamers. Am. J. Pathol..

[110] Hicke B.J., Marion C., Chang Y.F., Gould T., Lynott C.K., Parma D., Schmidt P.G., Warren S. (2001). Tenascin-C aptamers are generated using tumor cells and purified protein. J. Biol. Chem..

[111] Lindner V., Giachelli C.M., Schwartz S.M., Reidy M.A. (1995). A subpopulation of smooth muscle cells in injured rat arteries expresses platelet-derived growth factor-B chain mRNA. Circ. Res..

[112] Iida H., Seifert R., Alpers C.E., Gronwald R.G., Phillips P.E., Pritzl P., Gordon K., Gown A.M., Ross R., Bowen-Pope D.F., Johnson R.J. (1991). Platelet-derived growth factor (PDGF) and PDGF receptor are induced in mesangial proliferative nephritis in the rat. Proc. Natl. Acad. Sci. USA.

[113] Heldin C.H. (1992). Structural and functional studies on platelet-derived growth factor. EMBO J..

[114] Green L.S., Jellinek D., Jenison R., Ostman A., Heldin C.H., Janjic N. (1996). Inhibitory DNA ligands to platelet-derived growth factor B-chain. Biochemistry.

[115] Morishita R., Sugimoto T., Aoki M., Kida I., Tomita N., Moriguchi A., Maeda K., Sawa Y., Kaneda Y., Higaki J., Ogihara T. (1997). *In vivo* transfection of cis element "decoy" against nuclear factor-kappaB binding site prevents myocardial infarction. Nat. Med..

[116] Ostendorf T., Kunter U., Grone H.J., Bahlmann F., Kawachi H., Shimizu F., Koch K.M., Janjic N., Floege J. (2001). Specific antagonism of PDGF prevents renal scarring in experimental glomerulonephritis. J. Am. Soc. Nephrol..

[117] Ostendorf T., Kunter U., van Roeyen C., Dooley S., Janjic N., Ruckman J., Eitner F., Floege J. (2002). The effects of platelet-derived growth factor antagonism in experimental glomerulonephritis are independent of the transforming growth factor-beta system. J. Am. Soc. Nephrol..

[118] Jain R.K. (1987). Transport of molecules in the tumor interstitium: a review. Cancer Res..

[119] Jain R.K. (1996). Delivery of molecular medicine to solid tumors. Science.

[120] Pietras K., Ostman A., Sjoquist M., Buchdunger E., Reed R.K., Heldin C.H., Rubin K. (2001). Inhibition of platelet-derived growth factor receptors reduces interstitial hypertension and increases transcapillary transport in tumors. Cancer Res..

[121] Ni Z., Hui P. (2009). Emerging pharmacologic therapies for wet age-related macular degeneration. Ophthalmologica.

[122] Hunter T. (1993). Braking the cycle. Cell.

[123] Nevins J.R. (1992). E2F: a link between the Rb tumor suppressor protein and viral oncoproteins. Science.

[124] Giangrande P.H., Zhang J., Tanner A., Eckhart A.D., Rempel R.E., Andrechek E.R., Layzer J.M., Keys J.R., Hagen P.O., Nevins J.R., Koch W.J., Sullenger B.A. (2007). Distinct roles of E2F proteins in vascular smooth muscle cell proliferation and intimal hyperplasia. Proc. Natl. Acad. Sci. USA.

[125] Ishizaki J., Nevins J.R., Sullenger B.A. (1996). Inhibition of cell proliferation by an RNA ligand that selectively blocks E2F function. Nat. Med..

[126] Rusconi C.P., Yeh A., Lyerly H.K., Lawson J.H., Sullenger B.A. (2000). Blocking the initiation of coagulation by RNA aptamers to factor VIIa. Thromb. Haemost..

[127] Rusconi C.P., Scardino E., Layzer J., Pitoc G.A., Ortel T.L., Monroe D., Sullenger B.A. (2002). RNA aptamers as reversible antagonists of coagulation factor IXa. Nature.

[128] Rusconi C.P., Roberts J.D., Pitoc G.A., Nimjee S.M., White R.R., Quick G., Scardino E., Fay W.P., Sullenger B.A. (2004). Antidote-mediated control of an anticoagulant aptamer *in vivo*. Nat. Biotechnol..

[129] Becker R.C., Povsic T., Cohen M.G., Rusconi C.P., Sullenger B. (2010). Nucleic acid aptamers as antithrombotic agents: Opportunities in extracellular therapeutics. Thromb. Haemost..

[130] Blann A. (1993). von Willebrand factor and the endothelium in vascular disease. Br. J. Biomed. Sci..

[131] Verma I.M., Stevenson J.K., Schwarz E.M., Van Antwerp D., Miyamoto S. (1995). Rel/NF-kappa B/I kappa B family: intimate tales of association and dissociation. Genes Dev..

[132] Sawa Y., Morishita R., Suzuki K., Kagisaki K., Kaneda Y., Maeda K., Kadoba K., Matsuda H. (1997). A novel strategy for myocardial protection using *in vivo* transfection of cis element 'decoy' against NFkappaB binding site: evidence for a role of NFkappaB in ischemia-reperfusion injury. Circulation.

[133] Tomita T., Takano H., Tomita N., Morishita R., Kaneko M., Shi K., Takahi K., Nakase T., Kaneda Y., Yoshikawa H., Ochi T. (2000). Transcription factor decoy for NFkappaB inhibits cytokine and adhesion molecule expressions in synovial cells derived from rheumatoid arthritis. Rheumatology (Oxford).

[134] Ueno T., Sawa Y., Kitagawa-Sakakida S., Nishimura M., Morishita R., Kaneda Y., Kohmura E., Yoshimine T., Matsuda H. (2001). Nuclear factor-kappa B decoy attenuates neuronal damage after global brain ischemia: a future strategy for brain protection during circulatory arrest. J. Thorac. Cardiovasc. Surg..

[135] Barfod A., Persson T., Lindh J. (2009). *In vitro* selection of RNA aptamers against a conserved region of the Plasmodium falciparum erythrocyte membrane protein 1. Parasitol. Res..

[136] Unwalla H.J., Li H., Li S.Y., Abad D., Rossi J.J. (2008). Use of a U16 snoRNA-containing ribozyme library to identify ribozyme targets in HIV-1. Mol. Ther..

[137] Sriram B., Banerjea A.C. (2000). *In vitro*-selected RNA cleaving DNA enzymes from a combinatorial library are potent inhibitors of HIV-1 gene expression. Biochem. J..

[138] Tuerk C., MacDougal S., Gold L. (1992). RNA pseudoknots that inhibit human immunodeficiency virus type 1 reverse transcriptase. Proc. Natl. Acad. Sci. USA.

[139] Burke D.H., Scates L., Andrews K., Gold L. (1996). Bent pseudoknots and novel RNA inhibitors of type 1 human immunodeficiency virus (HIV-1) reverse transcriptase. J. Mol. Biol..

[140] Khati M., Schuman M., Ibrahim J., Sattentau Q., Gordon S., James W. (2003). Neutralization of infectivity of diverse R5 clinical isolates of human immunodeficiency virus type 1 by gp120-binding 2'F-RNA aptamers. J. Virol..

[141] Kanamori H., Yuhashi K., Uchiyama Y., Kodama T., Ohnishi S. (2009). *In vitro* selection of RNA aptamers that bind the RNA-dependent RNA polymerase of hepatitis C virus: a possible role of GC-rich RNA motifs in NS5B binding. Virology.

[142] Jang K.J., Lee N.R., Yeo W.S., Jeong Y.J., Kim D.E. (2008). Isolation of inhibitory RNA aptamers against severe acute respiratory syndrome (SARS) coronavirus NTPase/Helicase. Biochem. Biophys. Res. Commun..

[143] Gopinath S.C., Sakamaki Y., Kawasaki K., Kumar P.K. (2006). An efficient RNA aptamer against human influenza B virus hemagglutinin. J. Biochem..

[144] Jing N., Marchand C., Liu J., Mitra R., Hogan M.E., Pommier Y. (2000). Mechanism of inhibition of HIV-1 integrase by G-tetrad-forming oligonucleotides *in vitro*. J. Biol. Chem..

[145] Jing N., Hogan M.E. (1998). Structure-activity of tetrad-forming oligonucleotides as a potent anti-HIV therapeutic drug. J. Biol. Chem..

[146] Bellecave P., Cazenave C., Rumi J., Staedel C., Cosnefroy O., Andreola M.L., Ventura M., Tarrago-Litvak L., Astier-Gin T. (2008). Inhibition of hepatitis C virus (HCV) RNA polymerase by DNA aptamers: mechanism of inhibition of *in vitro* RNA synthesis and effect on HCV-infected cells. Antimicrob. Agents Chemother..

[147] Duconge F., Toulme J.J. (1999). *In vitro* selection identifies key determinants for loop-loop interactions: RNA aptamers selective for the TAR RNA element of HIV-1. RNA.

[148] Kolb G., Reigadas S., Castanotto D., Faure A., Ventura M., Rossi J.J., Toulme J.J. (2006). Endogenous expression of an anti-TAR aptamer reduces HIV-1 replication. RNA Biol..

[149] Boucard D., Toulme J.J., Di Primo C. (2006). Bimodal loop-loop interactions increase the affinity of RNA aptamers for HIV-1 RNA structures. Biochemistry.

[150] Aldaz-Carroll L., Tallet B., Dausse E., Yurchenko L., Toulme J.J. (2002). Apical loop-internal loop interactions: a new RNA-RNA recognition motif identified through *in vitro* selection against RNA hairpins of the hepatitis C virus mRNA. Biochemistry.

[151] Kikuchi K., Umehara T., Fukuda K., Hwang J., Kuno A., Hasegawa T., Nishikawa S. (2003). RNA aptamers targeted to domain II of hepatitis C virus IRES that bind to its apical loop region. J. Biochem..

[152] Kikuchi K., Umehara T., Fukuda K., Kuno A., Hasegawa T., Nishikawa S. (2005). A hepatitis C virus (HCV) internal ribosome entry site (IRES) domain III-IV-targeted aptamer inhibits translation by binding to an apical loop of domain IIId. Nucleic Acids Res..

[153] Kikuchi K., Umehara T., Nishikawa F., Fukuda K., Hasegawa T., Nishikawa S. (2009). Increased inhibitory ability of conjugated RNA aptamers against the HCV IRES. Biochem. Biophys. Res. Commun..

[154] Konno K., Fujita S., Iizuka M., Nishikawa S., Hasegawa T., Fukuda K. (2008). Isolation and characterization of RNA aptamers specific for the HCV minus-IRES domain I. Nucleic Acids Symp Ser (Oxf).

[155] Romero-Lopez C., Barroso-delJesus A., Puerta-Fernandez E., Berzal-Herranz A. (2005). Interfering with hepatitis C virus IRES activity using RNA molecules identified by a novel *in vitro* selection method. Biol. Chem..

[156] Romero-Lopez C., Diaz-Gonzalez R., Berzal-Herranz A. (2007). Inhibition of hepatitis C virus internal ribosome entry site-mediated translation by an RNA targeting the conserved IIIf domain. Cell Mol. Life Sci..

[157] Romero-Lopez C., Diaz-Gonzalez R., Barroso-delJesus A., Berzal-Herranz A. (2009). Inhibition of hepatitis C virus replication and internal ribosome entry site-dependent translation by an RNA molecule. J. Gen. Virol..

[158] Fan S., Wu F., Martiniuk F., Hale M.L., Ellington A.D., Tchou-Wong K.M. (2008). Protective effects of anti-ricin A-chain RNA aptamer against ricin toxicity. World J. Gastroenterol..

[159] Ulrich H., Ippolito J.E., Pagan O.R., Eterovic V.A., Hann R.M., Shi H., Lis J.T., Eldefrawi M.E., Hess G.P. (1998). *In vitro* selection of RNA molecules that displace cocaine from the membrane-bound nicotinic acetylcholine receptor. Proc. Natl. Acad. Sci. USA.

[160] Hess G.P., Ulrich H., Breitinger H.G., Niu L., Gameiro A.M., Grewer C., Srivastava S., Ippolito J.E., Lee S.M., Jayaraman V., Coombs S.E. (2000). Mechanism-based discovery of ligands that counteract inhibition of the nicotinic acetylcholine receptor by cocaine and MK-801. Proc. Natl. Acad. Sci. USA.

[161] Sivaprakasam K., Pagan O.R., Hess G.P. (2010). Minimal RNA aptamer sequences that can inhibit or alleviate noncompetitive inhibition of the muscle-type nicotinic acetylcholine receptor. J. Membr. Biol..

[162] Daniels D.A., Chen H., Hicke B.J., Swiderek K.M., Gold L. (2003). A tenascin-C aptamer identified by tumor cell SELEX: systematic evolution of ligands by exponential enrichment. Proc. Natl. Acad Sci. USA.

[163] Hicke B.J., Stephens A.W., Gould T., Chang Y.F., Lynott C.K., Heil J., Borkowski S., Hilger C.S., Cook G., Warren S., Schmidt P.G. (2006). Tumor targeting by an aptamer. J. Nucl. Med..

[164] Lupold S.E., Hicke B.J., Lin Y., Coffey D.S. (2002). Identification and characterization of nuclease-stabilized RNA molecules that bind human prostate cancer cells via the prostate-specific membrane antigen. Cancer Res..

[165] McNamara J.O., Andrechek E.R., Wang Y., Viles K.D., Rempel R.E., Gilboa E., Sullenger B.A., Giangrande P.H. (2006). Cell type-specific delivery of siRNAs with aptamer-siRNA chimeras. Nat. Biotechnol..

[166] Wullner U., Neef I., Eller A., Kleines M., Tur M.K., Barth S. (2008). Cell-specific induction of apoptosis by rationally designed bivalent aptamer-siRNA transcripts silencing eukaryotic elongation factor 2. Curr. Cancer Drug Targets.

[167] Bagalkot V., Farokhzad O.C., Langer R., Jon S. (2006). An aptamer-doxorubicin physical conjugate as a novel targeted drug-delivery platform. Angew. Chem. Int. Ed. Engl..

[168] Chu T.C., Marks J.W., Lavery L.A., Faulkner S., Rosenblum M.G., Ellington A.D., Levy M. (2006). Aptamer:toxin conjugates that specifically target prostate tumor cells. Cancer Res..

[169] Farokhzad O.C., Cheng J., Teply B.A., Sherifi I., Jon S., Kantoff P.W., Richie J.P., Langer R. (2006). Targeted nanoparticle-aptamer bioconjugates for cancer chemotherapy *in vivo*. Proc. Natl. Acad. Sci. USA.

[170] Cheng J., Teply B.A., Sherifi I., Sung J., Luther G., Gu F.X., Levy-Nissenbaum E., Radovic-Moreno A.F., Langer R., Farokhzad O.C. (2007). Formulation of functionalized PLGA-PEG nanoparticles for *in vivo* targeted drug delivery. Biomaterials.

[171] Dhar S., Gu F.X., Langer R., Farokhzad O.C., Lippard S.J. (2008). Targeted delivery of cisplatin to prostate cancer cells by aptamer functionalized Pt(IV) prodrug-PLGA-PEG nanoparticles. Proc. Natl. Acad. Sci. USA.

[172] Zhou J., Li H., Li S., Zaia J., Rossi J.J. (2008). Novel dual inhibitory function aptamer-siRNA delivery system for HIV-1 therapy. Mol. Ther..

[173] Zhou J., Swiderski P., Li H., Zhang J., Neff C.P., Akkina R., Rossi J.J. (2009). Selection, characterization and application of new RNA HIV gp 120 aptamers for facile delivery of Dicer substrate siRNAs into HIV infected cells. Nucleic Acids Res..

